# Development of Mobile App for Breast Examination Awareness Using Health Belief Model: A Qualitative Study

**DOI:** 10.31557/APJCP.2021.22.10.3151

**Published:** 2021-10

**Authors:** Arryana Nasution, Azlina Yusuf, Soon Lean Keng, Nur Syahmina Rasudin, Yulita Hanum P Iskandar, Imi Sairi Ab Hadi

**Affiliations:** 1 *School of Health Sciences, Universiti Sains Malaysia, Kubang Kerian, Kelantan, Malaysia. *; 2 *School of Medical and Life Sciences, Sunway University, Petaling Jaya, Selangor, Malaysia. *; 3 *Graduate School of Business, Universiti Sains Malaysia, Penang, Malaysia.*; 4 *Department of Surgery, Hospital Raja Perempuan Zainab II, Kota Bharu, Kelantan, Malaysia. *

**Keywords:** Mobile app, breast examination awareness, health belief model, breast cancer, breast health

## Abstract

**Background:**

Mobile health technologies are widely being used for delivering health behaviour interventions. However, there is insufficient evidence that they are integrating theory and only a few researchers utilized a qualitative approach in their study.

**Objectives:**

This paper aims to identify requirements in developing a breast examination awareness mobile app based on the component of the Health Belief Model (HBM) for integration in health promotion strategy.

**Methods:**

A qualitative approach using semi-structured in-depth interview was utilized in this study. A purposive sampling method was conducted among public women attending hospital services, software and content experts in a tertiary teaching hospital in the East coast of Peninsular Malaysia. These interviews were recorded, transcribed and organized using NVIVO 11. The main themes were identified through thematic analysis of the interview transcripts.

**Results:**

A total of 37 participants recruited in this study. The themes that emerged from the analysis are vulnerability, forecasting, reactive, influence, outcome and obstacles. The sub-themes findings supported the HBM’s component in terms of the requirement for are an infographic risk factor, video (symptoms, self-examination), info (metastasis, survival, screening, triple assessment, treatment, myth and facts, benefit of early treatment, support groups), features (screening reminder, sharing button, prompt) and mobile app’s design.

**Conclusion:**

The research findings could provide a guide for future app development from public women, content and software experts. The information will be used to develop a breast examination awareness mobile app integrated with health theories.

## Introduction

In Malaysia, breast cancer (BC) is the most common cancer among women and it is estimated that the lifetime risk is one for every 27 female (NCI, 2019). Besides, the number of women diagnosed with late-stage BC (Stage III or IV) were increasing from 43.2% in 2007 to 2011 to 47.9% from 2012 to 2016 (Cheng et al., 2015; NCI, 2019). BC awareness among Malaysian women are also reported as poor (Ghazi et al., 2020; M. Lee et al., 2019) and the distributing factors are associated with educational background, social taboo, family history of BC and ineffective promotion (Ghazi et al., 2020; M. Lee et al., 2019). Breast examination awareness is defined as a technique to examine breast performed monthly by inspection and palpation and become familiar with their breast to recognise abnormalities (Thornton and Ram, 2008). Facilitation and intervention in breast examination awareness aim to increase awareness among Malaysian women. The common methods of delivery are through audio, video, letter, brochure, booklet, telephone counselling, newsletter, workshop, broadcast, lecture, electronic teaching aids, handouts (Austoker et al., 2009; Ifediora and Azuike, 2019; You, 2016), WhatsApp group (Pereira et al., 2020) and smartphone apps (Ribeiro et al., 2017).

 There has been an increasing interest in breast cancer mobile apps that focus on secondary and tertiary breast cancer prevention (Houghton et al., 2019). This technology has the potential to promote behaviour change (Kalke et al., 2020). However, there is lacking study that these mobile apps are integrating theory and utilized a qualitative approach to design and develop apps (Houghton et al., 2019; Kalke et al., 2020). A study by Ginossar et al., 2017 reported a gap in the expectation for BC apps and suggested that researchers need to identify user expectations for BC apps. In addition, the currently available mobile apps on breast cancer were lack the involvement of medical professionals, personalized (Mobasheri et al., 2014) and contextualized elements based on the local culture (Norsa’adah et al., 2012). A systematic review of the BC care apps study by Jongerius et al., (2019) found that most of the apps focused on BC management. Meanwhile, Kalke et al., (2020) revealed that most of the apps focused on the BC symptoms and breast self-examination (BSE) guidance. Other delivery method studies that employed HBM as a theoretical framework are cervical and breast cancer awareness screening (Kolutek et al., 2016; Leila et al., 2015), breast self-examination (Aghamolaei et al., 2011; Lu, 2001; Parsa et al., 2016). 

Along with the growth of mobile health technologies, however, there is increasing concern over mobile app performance limited by app design, accessibility, accuracy and data privacy (Chib and Lin, 2018). The challenges arise in designing for the ease of use of the mobile app, including considering the app’s accessibility, which affects the end-user acceptance of the app (Birkhoff and Moriarty, 2020; Fitzgerald and McClelland, 2017). Moreover, the app data developed and involving the health professional or academician are more relevant and reliable (Fitzgerald and McClelland, 2017). Meanwhile, the app’s effectiveness could be limited by the lack of theory-based intervention and insufficient tailored content (Naz et al., 2018; Ribeiro et al., 2017). Mobile app with tailored contents according to end-user requirement such as public women probably could help in their acceptance and effectiveness of the intervention (Lau et al., 2018). 

This paper aims to present a qualitative exploration of requirements and tailored information involving the user and the experts. The finding will be used to develop a theory-based breast awareness mobile app. The development of content and design is based on the component of the Health Belief Model (HBM). Mobile app development with integrating theory could stress and sustain health promotion behaviour change (Curtis et al., 2015; Mummah et al., 2016). The mobile app developed will be used in health promotion for public women. Therefore, improving the aesthetic appeal, user-friendly, acceptability and effectiveness of the mobile app is needed. 

## Materials and Methods

*Ethics*

Ethical approval for this study was obtained from the Human Research Ethics Committee (HREC) of Universiti Sains Malaysia (USM/JEPeM/18080380). Participants were informed about the study purpose and procedures by providing the information sheet. Autonomous right to participate in the study and confidentiality of the subject data were informed to the participants. Written informed consent was obtained from each participant before participating in the study.

*Study design*

The study utilized a descriptive qualitative method with individual, face-to-face, semi-structured in-depth interviews among public women, software experts and content experts to identify the requirement. A descriptive qualitative narrative describes a complete overview of the phenomenon (Kim et al., 2017). This qualitative method is used to gain insights into mobile app development and design (Francese et al., 2017; Lau et al., 2018). 

The interview guide was constructed based on the HBM domain: perceived susceptibility, perceived severity, perceived threat, cues to action, perceived benefit and perceived barriers. The interview guides provide features and contents based on the HBM component. HBM was chosen because it was proven by several studies to be effective in behaviour intervention through awareness (Akhtari-Zavare et al., 2016; El-kest et al., 2021; Keshavarz et al., 2020). The interviews were recorded, transcribed and organized using NVIVO 11 software.

*Sample and setting*

Purposive sampling was used in recruiting all three groups of participants. A total of 37 participants who fulfilled the inclusion and exclusion criteria were interviewed. Twenty-five participants were public women attending hospital services in a tertiary teaching hospital on the East coast of Peninsular Malaysia. As this study was parts of a bigger study, this setting was chosen as the conduct of the whole study was in the tertiary teaching hospital on the East coast of Peninsular Malaysia. Public women were selected based on the following inclusion criteria: (1) Female, age 18 years old and above; (2) owns a smartphone. Public women were excluded if self-reported mobile app literacy is low. Low mobile app literacy is defined as the inability to find, use, understand and evaluate the apps (Ginossar et al., 2017).

Six participants were software experts recruited from the Centre for Knowledge, Communication and Technology in the public university located on the east coast of Peninsular Malaysia. The software experts were selected based on their experience in software design. The software experts were excluded if their working experience is less than two years as only two years and more was regards as competent worker in their field (Mortensen et al., 2019).

Meanwhile, six participants were content experts recruited from the teaching hospital located on the east coast of Peninsular Malaysia. The content experts were healthcare professionals selected based on their BC patient’s management (breast surgeon, an oncology nurse and oncology counsellor). The content experts were excluded if their working experience is less than two years as only two years and more was regards as competent worker in their field (Mortensen et al., 2019).

*Data collection*

Data collection was conducted from April 2019 to July 2019 after obtaining ethical approval from the Human Research Ethics Committee of USM (HREC) and relevant authorities. The interview was conducted according to the participant’s preference time, date and location. A semi-structured interview guide (Table 1) was used to identify the main requirements of the breast awareness mobile app. The interview questions were topic based on the HBM domain used to guide the researcher (Ogden and Cornwell, 2010). The interview lasted for 30-45 minutes. All the interviews were audio-recorded and field notes were taken. Thematic saturation was reached at the 25^th^ interviews for public women, 6th interviews for content experts and 6th interviews for software experts.

*Data analysis*

We adopted the HBM component as the themes for the interview guide. All the interviews were audio-recorded. The first step was transcribed verbatim using thematic analysis methods. All the individual interview transcripts were transcribed into an Excel spreadsheet. The second step is generating a primary code to help guide the analysis by identifying similar categorized codes. The third step was followed by rereading and establishing the suitable themes, and the redundancies were removed. Explanatory verbatim quotes were selected cautiously to maintain data validity following the themes. 

## Results

A total of 48 eligible people were invited to participate. Eleven public women could not join due to a busy schedule or not interested in joining after explaining the study. A total of 37 agreed to participate in the study (response rate = 77.0%). Twenty-five public women attending hospital services, six software experts were from the Centre for Knowledge, Communication and Technology and six content experts in a tertiary teaching hospital in the East Coast of Peninsular Malaysia. The initials start with P represent public women, S represent software experts, and C represents content experts. Table 2 presents a summary of the socio-demographic and characteristics of all participants. 

The main results described based on HBM components supported by participants’ statement.

*Theme 1: Vulnerability*

All three groups were asked about risk factors and symptoms of BC topic. The questions asked for P group to explore what they know about the topic. The C group were asked to list down the topic to describe the scope of content that will be covered by the researcher or any contents that might need to be highlighted in the mobile app. The S group were asked about what they know and to describe the best way to present the content in the mobile app. 

*Subtheme 1.1: An Infographic risk factor*

Most of the public women were able to identify few risk factors of BC and however unable to state them. Some of the public indicated as follows:

“*Obesity, dense breast and what else? If she smokes, it will increase the risk factor. Hormone imbalance, such as high or low estrogen. That’s it. The estrogen hormone is an imbalance. (Laugh) I don’t remember.*” [Participant PAA]

“*For example, if we sleep at night wearing a bra is one of the factors. I’m not sure about the rest. That is the only one I know.*” [Participant PCA]

“*The simple one because if it’s too long, sometimes the one related to disease with include medical jargon and sometimes people felt that it’s it boring to read. So, we just give the simple one*” [Participant PSHM]

Some of the content experts in this study mentioned female gender, increasing age, prolonged estrogen exposure, low parity, first birth before 30 years old, smoking, alcohol intake, breastfeeding, family history and genetics are the risk factors of BC. The risk factors mentioned by the content experts are considered to be included as the content of the mobile application. They expressed their answers as follows:

“*Female, age, exposure to estrogen, low parity, and completion of first pregnancy more than 30 years of age is risk factors, alcohol, smoking, no breastfeeding.*” [Participant CMM]

“*Risk factors of breast cancer are mostly among patient with exposure to estrogen, for example, either they consume OCP or hormone replacement therapy. Mostly those with a family history have it strong we would say. Higher risk if the patient has family who had breast cancer, either mother or sisters. Close relative.*” [Participant CF]

“*We can’t just talk like this. Usually, the aunties will not understand. We usually used infographic video with cartoon images.*” [Participant CMI]

Meanwhile, the software experts’ suggested in the interview that the components of risk factors should be separated into two sections which are controllable and uncontrollable and use infographic format as it is easier for the user to refer and understand:

“*If it is controllable and uncontrollable means choose one, then followed by content for uncontrollable. Cannot make it left and right, definitely space will not be enough.*” [Participant SMN]

“For me, it is better in visual form and if possible, with images. It could alert and clearer for the user. In this context, perhaps each point for each image.” [Participant SZ]

*Subtheme 1.2: Video of BC’s symptoms*

Most public women were able to identify a lump as one of the BC symptoms. Talking about this issue, some participants mistook by mentioning the symptom of general cancer and the side effects of chemotherapy as the symptoms of BC:

“*Mass and lump around the breast, maybe discomfort around breast area such as pain when wearing a bra perhaps, or throbbing I guess or perhaps breast is usually soft become thicken, then the texture, I guess maybe the nipple is pulled in and then it is sore, then what else I have read. The breast can become sore, but this usually in the late stage, which already too late. It can be sore, puss, smelly, and it is exposed, bigger wound and what else. The symptoms usually fever, loss of appetite, vomiting, dizzy.*” [Participant PNNA]

“*From what I have read, there is lump, pain, tiring, sort of.*” [Participant PR]

“*Want to know the content, the symptoms for example. Is the skin shrink around it or is it painful in the breast or is it swelling all the time? That is the content that could guide us for self-checking.*” [Participant PR]

In the interview, the content experts mentioned BC symptoms, including a painless lump or symptomatic without any lump, which probably missed by the public women. This painless lump could be highlighted in the content. As reported in another study that awareness could enforce routine screening since someone could have cancer without having any symptom(Bashar et al., 2020):

“*Most come with a palpable lump. Most said cancer is painless right? It is painless because it manifest as a lump. Still, when we ask when you start having a symptom, she said it’s started with something very weak, discomfort which is sometimes you cannot differentiate between breast pain during menstruation. Once it already manifests with a lump, it is painless. It’s a painless lump.*” [Participant CJ]

“*Nipple discharge with colour like blood. It is compulsory to check for that. Our concern is that it could cause by cancer cell in the ductus*” [Participant CF]

The software experts’ suggestion in designing for the symptoms of BC is in the form of multimedia video with the combination of images and text to help the user understand the symptoms of BC. The BSE video, symptoms’ checkbox and pop-up ‘Go see the doctor!’ were designed on one page to alert the user while performing the examination. The pop-up alert of “*Go see the doctor!*” could be the cues to action for the mobile app’s user:

“*Multimedia video which combined image and text is clearer for the user*” [Participant SN]

 “*Such as alert for example if you have any symptoms, there’s a pop-up, please go see the doctor or please make an appointment, some sort like that*” [Participant SZ]

*Theme 2: Forecasting*

The next questions are based on the topic of those who are diagnosed with BC and what happened if it is not treated. The questions asked to the P group require them to describe their current understanding of the topic. The C group were asked the same questions as the P group and were asked specifically what are important information for the public to know regarding this topic. The researcher asked for suggestions from the S group on how will they design the mobile app for this topic. 

*Subtheme 2.1: Metastasis*

The public women mentioned it metastasize or spread to other parts of the body. Some of the women understand the process of metastasizing, and some are unable to describe, as she said, unsure of the process:

“*I think it will metastasis and spread to other organs too*” [Participant PAA]

“*Spread of course. I’m not sure physically how our breast would be. You know maybe there should be pus or how. Cancer we know that it can cause death*” [Participant PWK]

“*Show the photo of it spreading to make them feel a little scared.*” [Participant PNI]

Meanwhile, the content experts mentioned that the public should be aware of benign and metastasis cancer. One of the content experts also relates metastasis with BC staging and survival rate. Thus, the benign, metastasis, staging and survival rate will be included in the mobile application content:

“*For public information, we will state there are two types of cancer; one is called benign, second is metastasis.*” [Participant CTAD]

“*So in stage 4 which it metastasis distally, we labelled it as stage 4. Our option to treat the patient until she’s cured is less due to the low survival rate of stage 4*” [Participant CF]

The software expert suggested that the contents should be made in one display to simplify the straightforward explanation. Therefore, the staging of BC according to the TNM classification system was designed in infographic and table:

“*Ok, what are the symptoms for stage three or what are the effects to her, so all is already there in one display.*” [Participant SWMF]

“*It might be loading and slow. So, we make it in one display, simple! Use simple sentences such as tired, imbalance hormone. Use clear, straightforward sentences don’t need to explain everything.*” [Participant SWMF]

*Subtheme 2.2: Survival*

The public women in the interviews did mentioned and aware of death concerning BC. However, one participant also expressed that she is unsure about BC survivor:

“*Usually, did not get the early treatment, it can lead to death. Or did anyone survive?*” [Participant PCA]

“*It will burst if already stage 4 and followed by death.*” [Participant PNI]

Meanwhile, the content experts stated that even though the statistic of BC is increasing compared to ten years back, the survival rate is also increasing due to the increase of awareness. In the development of the mobile app, the currently available statistic of survival rate by stages, year and ethnicity were included:

“*According to breast cancer statistic, the cases are increasing but at the same time, the survival rate is also increasing because of awareness*” [Participant CTAD]

“*Currently it is about 1 in 8 women. Just to let others know that it is getting serious, the incident is more compared to ten years back. Then, highlight the survival statistic; we have a fixed five years survival rate for every stage*” [Participant CF]

One of the software experts also stated that the statistic format with appropriate information could have an impact on the users. 

“*Need to show the MAKNA cancer patient statistic according to the death rate, survivor or stage statistic perhaps. It depends on which can give an impact to the user, such as the current statistic which is appropriate in giving information.*” [Participant SZ]

*Theme 3: Reactive*

During the interview, the researcher questioned the P group on how to detect BC early and how they would like to have it presented in the mobile app. Besides, when the researcher asked the C group how to detect BC early, they shared their current practice and protocol with the public and the feedbacks of their current methods. Moreover, the S group also were asked what their suggestions are and how the presentation could help the mobile application users when using it.

*Subtheme 3.1: Video of BSE*

Public women have expressed that they prefer to know BSE steps with simple sentences. The user needs to know their normal breast and check for any abnormal symptoms. Video of BSE and the symptoms of BC were included in the mobile app as a reference for the users:

“*Explain steps by steps, like when we palpate and not sure is it a lump, normal or mammary gland. So maybe can explain how easy it is to identify the hardness of the lump*” [Participant PNA]

“*They have to teach, maybe can show video, it’s like if you’re going to the hospital they give pamphlet right? It had every photo with hand raised to reach the back and everything.*” [Participant PNI]

The current practices, as expressed by the content experts, are using infographic video and breast model mannequin to educate the public women. The BSE video in the mobile app could help the users continue to practice the correct technique of BSE on their own. A cartoon animation was also used in the mobile application development:

“*Usually, we will produce an infographic video that uses cartoon diagrams. Secondly, we will bring breast manikin that has a lump to show the normal and the abnormal. If it’s not normal, we put a marble inside. Detect, try to check carefully. It’s easier because it’s more towards the senses. As touches and observes is understandable even if the steps are wrong*” [Participant CMI]

“*Most of these people are not good at it and so when they come to the hospital at the stage with discharge or pain. There are symptoms showing breast cancer. They even claim to perform it but nothing.*” [Participant CTAD]

Even the software expert expressed that the animation should be steps-by-steps, using cartoon instead of a real person and marked the area which needs to be check so that it is clear for the users’ reference:

“*Do some animation to sample the object of the breast. Let’s make the animation change colour. It should be step-by-step.*” [Participant SZ]

“*Probably people are shy if the self-exam is using a human being, can use cartoon to show such things. It might be more interesting, and they are not shy to do it on their own*” [Participant SZ]

“*Keep in mind the one that needs to be check. So, the area areas need to be marked. Such as going to the gym, the affected area will be marked. *” [Participant SNS]

*Subtheme 3.2: Screening information and triple assessment.*

Some public women expressed that they knew about mammogram or BSE only. Resulting to add information on screening examination in the mobile app content:

 “*I only know mammogram.*” [Participant PAA]

 “*Early detection is that we were told to do our check-up.*” [Participant PR]

 “*Some said there is a mammogram at the hospital, but the waiting list is long.*” [Participant PNI]

Meanwhile, the interview with the content experts has mentioned mammogram, clinical breast examination and BSE. They also stated that triple assessment should be included to educate the public that it is necessary to diagnose for BC and not by physical assessment only. Thus, the researcher has included content on triple assessment in the mobile application:

 “*Screening such as mammogram and a professional examination by the doctor or nurse. Examination of the breast cannot detect an early tumour until the patient can palpate it before proceeding with the investigation. To detect early tumour is by mammogram at the age of 40,45 or 50.*” [Participant CMM]

 “*They usually asked the doctor either the lump is cancer or not. We wouldn’t know until we completed the triple assessment. Triple assessment is a mammogram or ultrasound with biopsy. Then, we can confirm.*” [Participant CMM]

Besides that, the P group were asked to tell what they know about the treatments for BC and what they want to be included in the mobile application. Similar to the C group who were asked to tell about the treatment of BC. Meanwhile, the S group were asked if they know about BC treatment and how would they suggest the layout of the BC treatment content.

*Subtheme 3.3: Information on the treatment of BC*

The public women responded by answering removal of breast, chemotherapy or radiotherapy when asked about treatment. Moreover, one of the public women has mistaken biopsy as the treatment of BC: 

“*As I said earlier, near the early stage, she may have the biopsy, second chemotherapy, that’s all. What people call it ra..ra..ra. (Participant could not recall the word radiotherapy)*” [Participant PWK]

“*The treatment is because of never been treated; they don’t know very well. I know most people do is the examination; the one that we do ourselves need to be done regularly.*” [Participant PCA]

 “*After the test and it is confirmed cancer, they will remove it followed by chemo then followed by radiotherapy. No more I think because my friend after chemo, radiotherapy and that’s all for her*” [Participant PNI]

“*First of all, the most important are the symptoms. For example, if they had symptoms, they will look for resources. Any place she can refer to, treatments option and then any group supports than could ease her sadness and anything else.*” [Participant PH]

The content experts mentioned surgery, chemotherapy and radiotherapy. Usually, the treatment management of the patient is decided by the team after the assessment is done. However, for the content development, brief information related to the treatment was included in the mobile application:

 “*The treatments are general surgery, chemo and radiotherapy. But to be specific, we follow the patient’s age, cancer size and cancer size but in general, we remove the primary tumour followed by chemotherapy and radiotherapy*” [Participant CF]

“*But even with chemo[therapy], we will inform that not all patients will undergo chemo. After the surgery, the doctor will consult the oncologist first. If the oncologist suggested chemo, the patient would go for chemo. However, patient even though they did not go for operation yet, still they will ask for chemo first.*” [Participant CF]

The software expert mentioned that the content on the treatment section should be developed with diagrams to make it more straightforward. Every explanation is constructed in the most specific sentences and point forms. The suggestion to include the steps-by-steps of the treatment was not included due to the different treatment planning according to individual assessment:

“*Then, each treatment can explain. Elaborate on every section separately.*” [Participant SMN]

“*Display such as typography or figures of surgery, the steps*” [Participant SN]

*Theme 4: Influence*

In the interview, the researcher queried all three groups on how would they like to be reminded by the mobile application about breast awareness.

*Subtheme 4.1: Screening reminder features*

The public women mentioned that they would prefer if the mobile app have a screening reminder feature that can remind them to do BSE, clinical breast examination or another appointment: 

 “*It would be great if it can remind us to do a self-examination or check-up at the hospital.*” [Participant PAA]

“*Busy people don’t remember. At least the reminder keep on reminding will trigger the user to do it.*” [Participant PNI]

The content expert also mentioned that the mobile app should have a reminder feature with a pop-up notification. This feature was included in the mobile application to remind the users: 

 “*Next check-up or anything. The reminder. Have you checked your breast, a notification will pop-up once a month*” [Participant CMI]

Even the software experts suggested for reminder notification feature which repeats once a month and has an option of snooze or alarm:

“*Notification in the apps itself. Then we can send SMS from the apps itself. It’s like push notification but send as SMS or telegram. However, SMS will be charged, if it is free, its telegram such as telegram boat.*” [Participant SMN]

“*Function like notification. We make a notification to remind her.*” [Participant SN]

“*Then, if we made a reminder that repeats every month, perhaps. This repeat should have the option either snooze or alarm.*” [Participant SNS]

*Subtheme 4.2: Sharing button*

Some public women mentioned the ‘share button’ as they were already familiar with the feature that is also available on Facebook. However, event updates are not included in the mobile app as it requires an admin to handle all the update:

“*Share it with your friends; that info is important. If we have info even if we don’t have breast cancer, we can share the symptoms. This, we can tell others.*” [Participant PNI]

“*Easy to share this information.*” [Participant PS]

“*Facebook have the share features which is good if can be included in the apps, let say if today there is free health check-up in USM so that we can share the one in the apps and they will know about it.*” [Participant PSHM]

The content expert focus on BSE and the researcher have included the sharing button for the users to share their knowledge and reference just by sharing with their family directly from their phone or by sharing the link to download the mobile app BrAware:

“*Focus on BSE, mammogram screening. If she knows BSE, she can share with her family and her sibling*” [Participant CF]

The sharing medium can be through Facebook and WhatsApp as expressed by the software expert:

“*Share it doesn’t matter in FB or WhatsApp, we can link with the button.*” [Participant SZ]

*Subtheme 4.3: Prompt*

During the interview, the public women suggested for the mobile app to be able to prompt if the user has any symptom:

 “*I think I have a lump. What should I do? Even other people probably don’t know what to do. Maybe if those apps explain if you have a lump and we can hit the button yes or no. So maybe if yes, where to go? Then, if no, go to the next page.*” [Participant PNA]

The content experts mentioned visual as a powerful tool, so we included images and videos containing BC symptoms with steps of BSE. The suggestion of OT room images was not included due to the probability that the photos will be misused: 

 “*For me, visual is a powerful way to educate people if they can’t understand the description.*” [Participant CJ]

“*The images we took only to be shown at that place only. We cannot show them to the public because people will use those pictures to offer their product. That’s why the images that we took in the OT room. We will bring if we’re on the move.*” [Participant CMI]

The software expert also suggested including the google maps feature to the nearest centre in the mobile app. Even though video could have more impact compared to text and images, it is mentioned by the software expert that too much video could make the mobile app to be heavy due to the file size. So in this mobile app, only one video was included:

 “*Following the latest technology using google maps in the Kota Bharu area, she can search if the apps have the features. Perhaps there she can refer from the centres*” [Participant SZ]

“*If there are symptoms, “act early” “please act early” “please act immediately” “go to the nearest health clinic” or “please do urgent check-up*” it’s like an alert to her. [Participant SZ]

“*Of course, video delivers more impact, but if too much video, it is heavy because video size is big. Animation such as cartoon, the animation is heavy compared to text but is less than a video*” [Participant SZ]

*Theme 5: Outcome*

The researcher also asked group P. C and S about what is the benefit of BC awareness and having a breast awareness mobile application. 

*Subtheme 5.1: The benefit of early treatment*

Public women mentioned in the interview conducted that early treatment could save a life. Even the content expert said that getting early treatment is awareness among women. Meanwhile, the software expert suggested providing a space to leave a message to book or call back from the hospital:

 “*There is still treatment by the doctor to save her for stage 1 and stage 2. If she knew earlier, probably the treatment is not as hardcore such as stage 4*” [Participant PR]

 “*The component for early treatment is the patient’s awareness. Patients are aware of how to detect any abnormality in the breast. We are doing a breast screening campaign and the most important thing is to know the normal body and if there is any abnormality, she could immediately go for check-up and confirmation.*” [Participant CF]

 “*Maybe a hospital or one place that she can send like a message to booked or call back. At least if you are embarrassed, the hospital will have the detail to call back*” [Participant SA]

*Subtheme 5.2: BC support group*

Some of the public women interviewed mentioned that they prefer to communicate with BC survivors due to their experience. The content experts also expressed this suggestion to include the contact number of BC survivors and BC support groups. Meanwhile, one of the software experts suggested having an open forum feature:

 “*I prefer a cancer patient who had survived which means she has experienced it herself.*” [Participant PH]

“*Maybe we can put an expert for them to call. She read but probably got the incorrect information or from misleading person or include the phone number of breast cancer survivor*” [Participant CTAD]

“*Another one is the support group. I think this one you can find such as HRPZ, USM Bestari. If we want all over Malaysia because other hospitals have their support group such as Putrajaya have their support group*” [Participant CF]

“*So just make it an open forum. So I will post for an opinion if someone responded, there is a reply. If no one responded there would be no response.*” [Participant SNS]

*Theme 6: Obstacle*

In the interview, the researcher asked all three groups what are the barrier to breast cancer. The C group were asked how they consult patient or the public who expressed their fear. The S group were asked if there is any layout or design that they could suggest for this topic.

*Subtheme 6.1: Fear of treatment*

The public women mentioned that she would fear if it is already late-stage and fear of treatment. So, information related to BC treatment should be included in the mobile app. 

“*Scared to know that it is already staged 4, it’s bad already if knew earlier scared of the treatment. Scared of doing mammogram and everything.*” [Participant PCA]

“*Some people, if they have a lump, they are afraid to go to the hospital, afraid that the doctor will do things because people always said that chemo is painful which causes hair loss.*” [Participant PNI]

Lacking awareness of management and the type of surgery of BC could contribute to women’s fear expressed by the content expert. So, the researcher has included information related to BC surgery to provide some insight to the users. 

“*It’s associated with losing one’s breast too. That’s probably one of the bittering factors that prevent them from coming. Thinking the doctor will remove it even though it’s not confirmed. If the size is small, we will remove the affected area only.*” [Participant CMM]

One of the software experts expressed to include steps by steps for treatment to create awareness:

“*Awareness for her to go for treatment perhaps. Maybe steps by steps for the first stage, maybe she’s afraid of it.*” [Participant SZ]

*Subtheme 6.2: Myths and facts*

In the interview session with the public women and content expert, the participants did mention the sharp object that can cause the spread of BC. Moreover, there is also an obstacle to getting early treatment, such as traditional healer or alternative medicine. Myths and facts could provide information for the users:

“*But some people said cancer is due to metal such as a knife to spread.*” [Participant PNI]

“*There may be some myths that sharp objects may cause it to be even worse. Our body has made a wall around it. When we did a biopsy, we have breached the wall. Some have documented publication that needle track would have seedling because if we did thru cut biopsy that vacuumed and causing pulled out and left behind. This is probably true; however, we need a biopsy to proceed with the treatment. Unlike if we did a biopsy and did not do anything, definitely it will get worse.*” [Participant CMM]

 “*The obstacle is alternative medicine because now alternative medicine is not only ‘bomoh’[traditional healer]. We used to think that ‘bomoh’ is the obstacle because patients seek ‘bomoh’ to avoid surgery, but now we have obstacles from modern alternative medicine such as stem cell and many more. *” [Participant CF]

“*Our concern is towards diagnosed women who cannot accept their condition. When they cannot accept it, they tend to do unnecessary thing such as seeking for ‘bomoh*’” [Participant SWMF]

Moreover, the researcher also asked if any barriers cause them to not use the mobile application. The S group were asked on this topic on how the researcher can improve the mobile application.

*Subtheme 6.3: Mobile app design*

Some public women mentioned that they are not interested in the mobile app if it is not interactive, complicated and not easy to access.

 “*Among the problems that turn me off is when the apps are primary school level. Have you ever used the JPA website, it’s under the government. When we touch, it’s not too interactive.*” [Participant PNA]

“*Make this app simple, easy for people to access; if it’s simple and appealing people will be interested.” [Participant PNI]*

For mobile app design, the software experts suggested using monographic and feminine colour. They also mentioned that the mobile app design should be in simple steps with big buttons, infographics, or the explanation should be in short sentences. They also suggested for side menu and limit the page scroll limit to three times the scroll maximum. Meanwhile, the experts told me not to use a weird font, and the font size can be customized to users’ preference. 

 “*What matters is the colour and font system itself. Like me, I would focus on design. If possible, don’t use a weird font, not striking colour, in many tones. There is a red colour, blue colour, yellow colour and then a green colour which is the Flintstones era; nowadays we use monographic colour.*” [Participant SMN]

“*If anyone doesn’t see it, they can enlarge it; we customise the font size.*” [Participant SNS]

“*Make it simple, suitable colour, probably feminine colour, one more is that not so many steps required. Big buttons, an infographic is important and avoid long essays.*” [Participant SWMF]

“*Make a menu, click on the side to access the menus, side menu, on top or whatever. Don’t put it on the pages which need to scroll, no need. What I remember is 3, 1..2. enough. (While scrolling on the phone screen). This is one, scroll one page 2, (scrolling) page 3, enough. Two times finger movements, so 3-page maximum.*” [Participant SWMF]

**Table 1:** Semi-Structured Interview Guide for an Individual Face-to-Face Interview

Domain	Topics
Perceived susceptibility	The risk factor(s) of breast cancer
	The symptom(s) of breast cancer
Perceived severity	Diagnosed with breast cancer
	Breast cancer if untreated
Perceived threat	Detect breast cancer early
	Breast cancer treatment
Cues to action	Reminded by the mobile application about breast awareness
Perceived benefit	The benefit of breast awareness
Perceived barriers	Barrier toward breast awareness

**Table 2:** Socio-Demographic Characteristic of All Participants in the Qualitative Interview (N=37)

Initial	Age	Ethnicity	Occupation	Highest education level	Household income (RM)	Family history of BC	Marital status
PNA	24	Malay	Research assistant	Degree	550	X	Single
PCA	53	Malay	Administrative assistant	SPM	4,000.00	X	Married
PAA	26	Malay	Student	Degree	10,000.00	√	Single
PR	34	Malay	Customer service executive	SPM	4,000.00	X	Single
PNI	50	Malay	Teacher	Degree	8,000.00	X	Married
PWK	25	Malay	Student	Degree	8,000.00	X	Single
PH	33	Malay	Housewife	Degree	3,000.00	X	Married
PNME	32	Malay	Beautician spa therapist	SPM	2,000.00	√	Single
PS	31	Siamese	Medical laboratory technician	Diploma	6,000.00	X	Married
PRI	32	Malay	Teacher	Degree	8,000.00	X	Married
PRA	52	Malay	Teacher	Degree	12,000.00	X	Married
PSHM	47	Malay	Teacher	Degree	5,000.00	X	Married
PRR	52	Malay	Bank clerk	SPM	4,700.00	X	Married
PSH	46	Malay	Teacher	Degree	10,000.00	√	Married
PSRR	21	Indian	Student	Matriculation	10,000.00	√	Single
PZ	29	Malay	Sale executive	MBA	2,500.00	√	Single
PNS	36	Malay	Housewife	Pra-Diploma	5,000.00	X	Married
PSHZ	18	Malay	Student	SPM	4,000.00	X	Single
PNSI	23	Malay	Student	Degree	1,000.00	X	Single
PSCA	47	Malay	Teacher	Degree	16,000.00	X	Married
PN	49	Malay	Manager	STPM	4,000.00	X	Single mom
PNH	41	Malay	Housewife	Degree	5,000.00	X	Married
PNSM	25	Malay	Graduated	Degree	3,000.00	√	Single
PRCH	52	Malay	Housewife	SPM	700	X	Married
PNNA	27	Malay	Self-employed	Degree	2,000.00	X	Single
SMN	38	Malay	IT officer	Degree	8,000.00	X	Married
SN	41	Malay	Assistant IT officer	Diploma	4,000.00	X	Married
SNS	40	Malay	IT officer	Degree	8,000.00	X	Married
SZ	48	Malay	IT officer	Master	8,000.00	X	Married
SWMF	35	Malay	IT officer	Degree	10,000.00	X	Married
SA	42	Malay	IT officer	Degree	6,000.00	√	Married
CJ	47	Malay	Lecturer	Master	30,000.00	X	Married
CMI	33	Malay	Administration officer	Master	5,000.00	X	Married
CMM	40	Malay	Doctor	Master	10,000.00	X	Married
CTAD	39	Malay	Lecturer	PhD	7,000.00	X	Married
CWZ	44	Malay	Surgeon	Master	15,000.00	X	Married
CF	46	Malay	Nurse	Diploma	5,000.00	X	Married

**Table 3:** Key Themes, Subthemes and Recommendations

Themes	Recommendation
Vulnerability	
Infographic risk factor	Separate section for the controllable and uncontrollable risk factor, add images, and state in point form.
Video of BC’s symptoms	Images and text, tick checkbox with a pop-up message “Go see the doctor!”
Forecasting	
Metastasis	Content included benign and metastasis, BC staging and survival rate. It is made in one display with a straightforward explanation.
Survival	Include currently available statistics of survival rate by stages.
Reactive	
Video of BSE	Step-by-step with a simple explanation. Infographic video. Cartoon instead of a human. Mark checking area.
Info Screening & triple assessment	Info on BC can be detected early by screening exam and confirmed by triple assessment.
BC treatment info	Elaborate in a section separately, figures or diagrams.
Influence	
Screening reminder	Pop-up, a notification, repeat once a month, the option to snooze and alarm.
Sharing button	Include sharing button to Facebook & WhatsApp.
Prompt	Prompt such as “Please do urgent check-up” if the user has symptoms. Add images and google maps features. video in the app should be limited.
Outcome	
The benefit of early treatment	Early treatment awareness. Access to book appointments.
BC support group	Include a contact number and open forum.
Obstacle	
Fear treatment	Content on treatment awareness.
Myth & facts	Facts to encounter myths perception.
Design criteria	Interactive, simple, easy access, monographic, feminine colour, big buttons, infographic, side menus, limit scrolling, standard font format and customise font size.

## Discussion

HBM components and public women guide this study, software experts and content experts’ involvement regarding their requirement and previous experience designing and developing a tailored breast awareness mobile app targeting public women. Together, these findings provide important insights to enhance breast examination awareness in public women using the mobile app. The end-user and experts’ involvement strategy ensures the mobile app acceptance and appropriateness, including selecting tailored content, design template, user interface, and features (Taymoori et al., 2020). A culturally and linguistically tailored content was proven to promote accessible health promotion and engagement towards BC screening (Davis and Oakley-Girvan, 2015; H. Lee et al., 2018; Wu et al., 2020). The development of the content has included some facts to encounter some cultural belief. Besides, the contents also were available in English and Malay. The study by H. Lee et al. 2018, even recommended navigation services to facilitate the users to go for BC screening. However, the google maps provided in our mobile application will navigate the users to the support groups according to states all over Malaysia. 

Generally, in theory, a higher perceived susceptibility, benefit and removal of barrier contribute to higher preventive action taken (Kissal and Kartal, 2019). Findings in this study revealed participants group P, C and S expressing the video’s requirement related to BC’s symptoms and BSE. Meanwhile, earlier studies with HBM based using video intervention has reported an increase in knowledge, confidence, self-exam intention, practice, ability to detect lump and reduce the barrier in seeking help (Kissal and Kartal, 2019; Parashar et al., 2020; Secginli and Nahcivan, 2011; Wahdi and Retno, 2020). In previous video intervention studies, topics included in the video are risk factors of BC, screening, BSE technique and skills (Ogletree et al., 2004; Shams et al., 2014; Wood et al., 2002). 

However, despite promising results from the video intervention used in many studies, the video content has not been optimized or standardized by competent authorities (Parashar et al., 2020). Thus, the involvement of the content and software expert in this study is an appropriate measure to corroborate the mobile app’s content for breast examination awareness among public women. From the qualitative research, the participant’s group S alluded to the notion of designing an infographic BC risk factor. Group P mentioned they preferred it to be simple and group C are already using infographic video. This finding was unexpected, and this result has not previously been described. There are, however, other studies that applied infographic design such as in cardiovascular disease risk factor and HIV-related patient (Oja et al., 2019; Stonbraker et al., 2019). This finding has an important implication for developing mobile app content in delivering a step-by-step or straightforward message visually (Go et al., 2020; Oja et al., 2019). Infographic intervention using symbols, images, cultural adaptation and local language could improve communication (Stonbraker et al., 2019) as it is easier for the public to understand health information (Go et al., 2020). Moreover, a study reported that their participants are not attracted to infographic design that is too complex or colourless (Harrison et al., 2015). 

The previous study has revealed the need to increase the awareness of BC’s perceived severity, which are defined as beliefs in health issues that will cause harm (Lotfi et al., 2012). When asked about perceived severity in this study, some of the participants’ group P, C and S mentioned metastasize and survival rate statistics. As the information on metastasis is different from other stages (Tucker et al., 2017) and even patient with metastatic BC also claimed that it is not easy to get it (Cardoso et al., 2018). We find it crucial to include information related to metastasis in the mobile app. The survival rate is affected by several factors, such as the time taken to diagnose, stage of the disease, treatment progress, access to care (Adam and Koranteng, 2020; Ssentongo et al., 2019). The user needs to understand the survival rate for them to act appropriately.

Prior studies have noted the importance of interventions to reduce time to diagnosis and avoid treatment delays (Setyowibowo et al., 2019). In this study, participants’ group P, C and S suggested including info on the screening exam, triple assessment and treatment options in the mobile app. This result is consistent with another study which requested for treatment and side effect to be included (Hou et al., 2020). In another study’s participants are also in agreement that knowledge related to treatment is helpful for them (Setyowibowo et al., 2019). Meanwhile, knowledge related to screening could decrease the death rate and cancer phases (Masoudiyekta et al., 2018). Hopefully, this perceived threat content will increase routine behaviour of performing BSE among the public women. As revealed in a study, those who never perform BSE are likely to have delayed examination (Wati et al., 2019). 

The participants’ group P, C and S mentioned the screening exam reminder, sharing button and prompt, and it is perceived as cues to action. In accordance with the present results, previous studies have demonstrated that the reminder system is an effective and acceptable intervention (Brown and Rahman, 2018; Heo et al., 2013; Sandhu et al., 2019). Meanwhile, a sharing button matches those observed in earlier studies for resource sharing, social engagement, providing support and promotion to others (Anderson et al., 2016; Baumel and Muench, 2016; Lapointe et al., 2013). Prompt usually used for goal setting, intention formation, self-monitoring and feedback in the mobile app (Kalke et al., 2020; Vollmer Dahlke et al., 2015). Moreover, a prompt would lead to user’s engagement, interaction and usage of the mobile app (Bidargaddi et al., 2018; Perski et al., 2017). 

As for the perceived benefit component, the participants’ group P and C expressed the need for early treatment and BC support groups. This rather interesting finding of support groups that offer emotional or spiritual helps social interaction, perspective, experience sharing, material and knowledge as mentioned in previous studies (Granado-Font et al., 2018; Hou et al., 2020; Kreps and Neuhauser, 2010). This result on early treatment further supports the idea of reducing morbidity or mortality rate (Ardahan et al., 2015), option for less aggressive treatment (Nur Aishah Taib et al., 2007) and improve survival rate (Rivera-Franco and Leon-Rodriguez, 2018). Another study revealed that an increase in perceived benefit and reduced barrier could lead to a change in users’ behaviour in BSE practice and mobile app usage (Mahmoud et al., 2018).

Acceptance towards mobile apps is contributed by the mobile app’s value and relevance (Fitzgerald and McClelland, 2017), such as in a Korean’s study where mobile app effectively promote BSE (Heo et al., 2013). Moreover, the findings related to the mobile app are in accord with an earlier study focusing on a design suggesting a preference for bigger fonts, colour scheme, scrollbars, buttons (Chib and Lin, 2018), easy browse, use images and plain language (Birkhoff and Moriarty, 2020; Ginossar et al., 2017). Furthermore, an interactive mobile app is the preferred one as it provides access to seek help, symptoms reporting and self-care (Crafoord et al., 2020; Gustavell et al., 2020). Besides that, most participants group P, C and S commented about the fear of treatment and myth. This result is in line with those in previous studies which stated fear of cancer treatment due to inadequate knowledge, suffer or fatalistic view of cancer, losing hope, expensive cost, side effects, ineffective treatment and believed that alternative treatments are more effective (Lim et al., 2015; Norsa’adah et al., 2012). This result also supports the previous research suggesting the need for education related to BC’s myths to reduce late diagnosis (Shamsi et al., 2020).

From the discussion, the voices of all the women and experts from the interviews are important to generate themes in this study. A qualitative approach used to gain insight from the interviews conducted (Francese et al., 2017; Houghton et al., 2019). A previous study reported physicians roles are important in promoting BC screening uptake but some of them encounter problems when patients do not know about BC screening (Wu et al., 2020). Hopefully, the developed mobile application based on the users’ requirements involving content and software experts would cater as a health promotion tools targeting breast examination awareness which include information related to BC detection and management.

*Limitation*

This study’s limitation is that the researchers only focus on mobile app content development based on the HBM component except for self-efficacy. Moreover, the present sample is purposive and small from one area in the tertiary teaching hospital thus it cannot be generalised to the other setting. 

*Further Research*

This research supported by a theory-based to guide the study, and further work is required based on the self-efficacy component such as to identify user’s experience and the ability to execute BSE routine using the mobile app. Besides, the researchers also suggested further exploration of expert consensus of the mobile application using a Fuzzy Delphi method for personalized learning tool. A focus group discussion for phase 2 also is recommended. 

In conclusion, this study explored breast examination awareness mobile app requirements among public women, software experts, and content experts. It is based on the HBM, a proven effective theory for behaviour intervention using a qualitative approach. The findings may guide the development in improving the app design and usability. A tailored-contents and features according to the participants’ need were designed exclusively. According to the HBM domain, the requirement was described, such as infographic risk factor, video (symptoms, self-examination), info (metastasis, survival, screening, triple assessment, treatment, myth and facts, benefit of early treatment, support groups), features (screening reminder, sharing button, prompt) and certain mobile app’s design criteria. 

## Author Contribution Statement

The authors confirm contribution to the paper as follows: Study conception and design: AY, SLK, YHPI; acquisition of data: AN; analysis and interpretation of the data: AN, AY, SLK, YHPI, ISAH; drafting manuscript: AN, AY, SLK, YHPI. All authors reviewed the results and approved the final version of the manuscript.
